# Noninvasive Evaluation of Liver Fibrosis in a Sample of Putative Inactive HBV Carriers in Rome, Italy

**DOI:** 10.1155/2021/3068690

**Published:** 2021-08-14

**Authors:** Marco Delle Monache, Alessio Petrelli, Alessandra Rossi, Roberto Cecere, Concetta Mirisola, Gianfranco Costanzo, Chiara Francia, Federica Cerini, Andrea Cavani, Lorenzo Nosotti

**Affiliations:** ^1^Liver and Infectious Diseases Unit, L. Parodi Delfino Hospital, ASL RM5 P.le A. Moro 1, Colleferro, Rome 00034, Italy; ^2^National Institute for Health, Migration and Poverty (NIHMP), Via di San Gallicano 25/a, Rome 00153, Italy

## Abstract

**Background:**

Noninvasive methods are useful for investigating patients with chronic HBV infection. The severity of liver disease in inactive HBsAg carriers can be noninvasively assessed by transient elastography (TE) alone or in association with biochemical markers of fibrosis.

**Objectives:**

The study evaluates the effectiveness of the TE compared to common fibrosis scores (FSs), APRI, Forns Index, and FIB4, for identifying significant fibrosis in Italian and foreigner HBsAg carriers. To investigate the risk of progression of the liver disease, liver stiffness (LS) and HBV-DNA were monitored over time.

**Methods:**

Viral load, biochemical parameters, and LS have been retrospectively evaluated in 125 putative inactive HBV carriers, who visited two outpatient departments (Colleferro Hospital and INMP) from 01/03/2014 to 31/12/2019. Differences in clinical, biochemical, and demographic variables between Italians and foreigners were analyzed. 66 of 125 patients were followed up for 24 months by monitoring liver stiffness and HBV-DNA.

**Results:**

Mean overall LS was 5.55 ± 1.92 kPa; 18 (14.4%) patients had a LS ≥7.5 kPa. Mean of APRI, Forns, and FIB4 was 0.29 ± 0.11, 4.15 ± 1.63, and 1.16 ± 0.59, respectively. FS did not differ between the patients with LS <7.5 kPa and those with LS ≥7.5 kPa. Italians displayed a significant lower ALT (0.53 ± 0.18 vs. 0.67 ± 0.33, *p* < 0.05) and AST (0.59 ± 0.16 vs. 0.70 ± 0.21, *p* < 0.01) value than foreigners. No differences in LS and HBV-DNA levels were observed. In 66 patients followed up for 24 months, HBV-DNA increased by ≥2000 UI/ml after 12 months in 15 individuals and remained ≥2000 UI/ml after 24 months in 10/15 individuals. 7/10 patients showed LS ≥ 7.5 kPa after 24 months, and 4 of them underwent antiviral therapy for HBV. Patients with HBV-DNA <2000 IU/ml had a significantly lower LS than those with HBV-DNA ≥2000 IU/ml (5.30 ± 1.43 vs. 7.69 ± 1.07, *p* < 0.0001).

**Conclusions:**

Analysis shows lower effectiveness of FS vs. TE in the assessment of putative inactive HBV carriers. Furthermore, using FibroScan® and HBV-DNA can identify “false” inactive carriers.

## 1. Background

Hepatitis B virus (HBV) infection is a worldwide health problem. It is estimated, in fact, that HBV in the world has infected 2 billion people and that there are more than 350 million chronic carriers of the virus. This infection is widespread in some Eastern European countries with a prevalence of 4.4% of the population [1]. In Italy, the overall prevalence of HBV was equal to 0.6% in 2016. In comparison, immigrants from East-Europe living in Italy showed HBsAg prevalence between 6.9% and 36.7% [2–5]. Infection by HBV can be responsible for liver diseases with different severities, from chronic hepatitis to cirrhosis and primary liver cancer. In asymptomatic patients, reactivation of the virus, i.e., a sudden increase in viral replication in previously inactive HBsAg carriers, is also possible and results in the development of chronic hepatitis.

In the past, liver biopsy (LB) was the gold standard for diagnosis and staging of chronic HBV liver disease. More recently, and according to the international guidelines [6, 7], inactive carriers of HBsAg are not subjected to invasive investigations, such as liver biopsy, but to noninvasive methods for the staging of liver disease, such as transient elastography (TE) alone or in association with fibrosis scores based on biochemical variables such as APRI, Forns Index, and FIB4 [6, 8–11]. These methods are easy, feasible, and well accepted by the patient and have sufficient accuracy [6]. Furthermore, according to some studies, they have similar results to LB and can be performed in patients of any age [6–10]. In the last two decades, noninvasive tests were used more frequently to evaluate patients with chronic HCV hepatitis [12] and nonalcoholic steatohepatitis (NASH) [13–18]. Furthermore, there is a good correlation between LS measured by FibroScan® and the stage of histological fibrosis in patients with HBV-positive cirrhosis [12, 13]. A meta-analysis [19] confirmed that TE has a good predictive value for advanced fibrosis (i.e., Metavir ≥ F2 and Ishak ≥ S3). Finally, TE of the latest generation (with CAP) can investigate other causes of liver injury [12, 20, 21], such as NAFLD. However, TE has drawbacks, the most significant of which is that LS can be affected by certain clinical conditions such as liver inflammation [20] and fatty liver infiltration [12].

## 2. Objectives

The principal aim of this retrospective study was to evaluate the effectiveness of TE compared to common biochemical fibrosis tests such as APRI, Forns Index, and FIB4 to rule out the presence of advanced fibrosis in presumed inactive carriers of HBsAg. Furthermore, we compared in our study population some clinical characteristics (age, ALT, AST, liver stiffness, and HBV-DNA) of Italians versus foreigner patients. Finally, we followed up a subgroup of patients to monitor liver stiffness and HBV-DNA levels.

## 3. Materials and Methods

### 3.1. Cohort Recruitment and End Points

We retrospectively assessed the medical records of 125 presumed inactive HBsAg carriers admitted to 2 outpatient departments (Colleferro Hospital and INMP) from March 2014 to December 2019. Inclusion criteria were age 18–65 years, HBsAg positivity for at least 12 months associated with normal ALT levels, and HBV-DNA less than 2000 IU/ml for at least one year.

### 3.2. Transient Elastography

LS was measured in all subjects. All TEs were performed using Fibroscan © (Echosens, Paris) by experienced doctors following the manufacturer's instructions. Patients fasted for at least two hours were examined in the dorsal decubitus position with the right arm in maximum abduction. The tip of the transducer was placed on the skin in the right intercostal spaces above the liver. LS mean value (kPa) was obtained from at least ten valid measurements excluding series with interquartile range >30% and success <60%. LS was analyzed as a continuous and categorical variable (<7.5 kPa, ≥7.5 kPa), through the value of 7.5 kPa considered as the cutoff for significant fibrosis in the HBV setting [11].

### 3.3. Histological and Biochemical Data

Liver histology, when available, was evaluated for the degree of necroinflammation and stage of fibrosis according to Ishak score [22].

HBV-DNA levels were determined by real-time PCR (Cobas® Taqman® 48 Roche Molecular Diagnostics), and quantitative HBsAg assay (qHBsAg) was performed using the Elecsys Quant® HBsAg II Roche assay. HBV-DNA was analyzed as a continuous and categorical variable (<2000 IU/ml, ≥2000 IU/ml), through the value of 2000 IU/ml considered as the cutoff.

Liver fibrosis scores were calculated by applying the corresponding formulas:

(1) $$\begin{array}{c} \text{APRI }=\;\frac{\text{AST}\left(\text{U}/\text{L}\right)/\text{AST}\left(\text{upper normal limit}\right)}{\text{platelet count}\left(10^{9}/\text{L}\right)\;}\times 100\left(7\right),\\ \text{FIB}-4\;=\frac{\text{age}\left(\text{years}\right)\;\times \text{ AST }\left(\text{U}/\text{L}\right)}{\text{platelet count}\left(10^{9}/\text{L}\right)\;\times \;\sqrt{\text{ALT }}\left(\text{U}/\text{L}\right)}\left(9\right),\\ \text{Forns Index}=7\text{.}811-3\text{.}131\text{ ln }\left(\text{PLT}\left(\frac{109}{\text{L}}\right)\right)+\text{ 0.}781\text{ ln}\left(\gamma \text{GT}\left(\text{IU}/\text{L}\right)\right)\\ +3\text{.}467\text{ ln}\left(\text{age}\left(y\right)\right)-\text{0.}014\left(\text{cholesterol}\left(\text{mg}/\text{dL}\right)\right)\left(10\right). \end{array}$$

The values of AST, ALT, and GGT are expressed as the ratio between the value and the upper limit of the laboratory range (AST and ALT: 40 IU; GGT 45 IU).

### 3.4. Clinical Management of Patients

**Figure 1:**
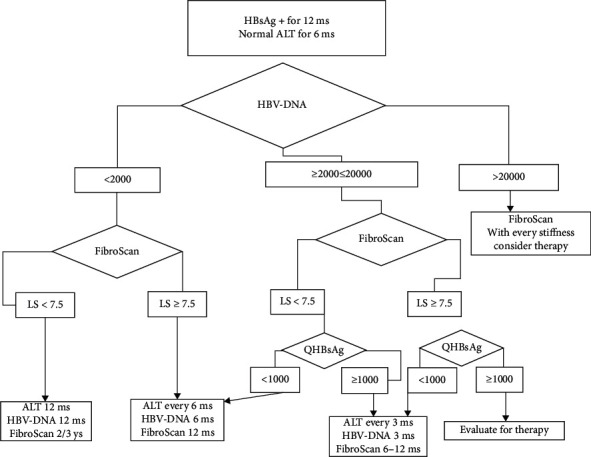
Clinical algorithm for the follow-up and management of putative inactive HBsAg carriers.

According to the EASL guidelines on the management of HBV infection [7], we applied a locally modified clinical flow chart in order to initiate antiviral therapy in patients with active infection and signs of liver fibrosis (Figure 1).

Sixty six patients were followed up for 24 months, and liver stiffness was measured every 12 months. During the follow-up, patients were differentiated according to HBV-DNA levels and LS: patients with DNA <2000 IU/ml and LS <7.5 kPa during the first year of follow-up were defined “true inactive” and monitored every 12 months for ALT, HBV-DNA, and LS every 2/3 years, while patients with LS ≥ 7.5 kPa were continued to be checked every 6 months for ALT, HBV-DNA, and LS every year. Patients who developed HBV-DNA ≥2000 and ≤20000 IU/ml during the follow-up, if LS was <7.5 and, when available, qHBsAg was <1000 IU/ml, were monitored with the same control intervals of the above. However, if qHBsAg was ≥1000 IU/ml, they were checked every 3 months for ALT and HBV-DNA and LS every 6–12 months. Patients with HBV-DNA ≥2000 and ≤20000 IU/ml, LS ≥7.5 kPa, and qHBsAg >1000 IU/ml underwent liver biopsy, if accepted, and began treatment if significant fibrosis was confirmed. Patients who developed during follow-up a HBV-DNA >20000 had a liver fibrosis evaluation and received antiviral treatment, regardless of liver stiffness.

### 3.5. Statistical Analysis

Baseline characteristics including demographical, biochemical, and liver elastographic variables of study population of 125 patients were presented as frequencies and mean ± standard deviation (SD). To analyze the existence of statistically significant differences in fibrosis scores according to liver stiffness (<7.5 kPa, ≥7.5 kPa), we performed the Wilcoxon test. We also tested the differences in clinical variables between Italians and foreigners through the Chi-square test and Wilcoxon test. Finally, in a subgroup of 66 patients followed up for 24 months, we analyzed the changes in HBV-DNA and liver stiffness during the follow-up through the Wilcoxon paired test and McNemar test and we tested the relation between HBV-DNA and liver stiffness through the Wilcoxon test and Fisher's test. We considered test results with *p* < 0.05 as statistically significant. All analyses were performed using statistical analysis software (SAS).

## 4. Results

Of the 125 patients enrolled, number of males was 77 (66%) and Italians was 75 (60%); the mean age was 46.5 years. Among foreigners, 27 (54%) were from countries of the Eastern Europe, mainly from Romania (*n* = 15, 30%), 18 (36%) from Africa, 4 (8%) from Asia, and 1 (2%) from South America.

In the study population of 125 patients, mean LS was 5.55 kPa (±1.92 SD); 18 (14.4%) patients had a LS ≥ 7.5 kPa. The mean value of APRI was 0.29 (±0.11 SD); the mean values of Forns Index and FIB4 score were 4.15 (±1.63 SD) and 1.16 (±0.59 SD), respectively (Table 1).

As shown in Table 2, Italians were significantly older than foreigners (mean ± SD: 53.1 ± 11.9 vs. 36.5 ± 10.2, *p* < 0.0001). Moreover, Italians, compared to foreigner patients, presented statistically significant lower values of ALT (mean ± SD: 0.53 ± 0.18 vs. 0.67 ± 0.33, *p* < 0.05) and AST (mean ± SD: 0.59 ± 0.16 vs. 0.70 ± 0.21, *p* < 0.01). Nevertheless, in both groups, the values of ALT and AST were in the normal range. No statistically significant differences were observed in LS and HBV-DNA distributions between Italians and foreigners.

Fibrosis score (APRI, Forns Index, and FIB4) distributions did not differ between patients with LS <7.5 kPa and those with LS ≥7.5 kPa (Table 3).

In the subgroup of 66 patients, we analyzed HBV-DNA and LS values at basal time, after 12 months and after 24 months. HBV-DNA increased to ≥2000 UI/ml after 12 months in 15 individuals and remained ≥2000 UI/ml after 24 months in 10 of 15 individuals. Seven of these 10 patients showed LS ≥7.5 kPa still after 24 months, and 4 of them, showing a moderate fibrosis (Ishak S ≥3) at liver biopsy, underwent antiviral therapy for HBV, and the other 3 patients refused liver biopsy and dropped out from the study.

Patients remaining with HBV-DNA<2000 IU/ml during the follow-up had a significantly lower LS distribution at 24 months than those with which HBV-DNA increased to ≥2000 IU/ml during the follow-up (mean ± SD: 5.30 ± 1.43 vs. 7.69 ± 1.07, *p* < 0.0001).

During the follow-up mean, HBV-DNA value increased significantly (mean ± SD: 561.80 ± 620.01 at basal time vs. 5202.76 ± 19597.25 at 24 months, *p* < 0.001), while mean LS value did not present significant change over time (mean ± SD: 5.80 ± 2.29 at basal time vs. 5.67 ± 1.62 at 24 months); patients with LS ≥7.5 kPa had a slight decrease from 16 at basal time to 12 after 2 years (Table 4).

Finally, in the subgroup of 66 patients followed for 24 months, we analyzed the relation between LS and HBV-DNA values: at basal time, all the patients presented HBV-DNA <2000 UI/ml and they had a LS mean distribution equal to 5.80 (±2.29 SD); at 12 months, LS values differed significantly between patients with HBV-DNA <2000 UI/ml (*n* = 51) and HBV-DNA ≥2000 UI/ml (*n* = 15) (5.33 ± 1.22 vs. 6.79 ± 1.55, *p* < 0.001); at 24 months, LS values differed significantly between patients with HBV-DNA <2000 UI/ml (*n* = 56) and HBV-DNA ≥2000 UI/ml (*n* = 10) (5.30 ± 1.43 vs. 6.79 ± 1.55, *p* < 0.0001) (Table 5).

## 5. Discussion

In our setting of putative inactive HBV carriers, the application of an easy and feasible algorithm coupling HBV-DNA and LS measurement allowed us to identify patients with a progressive liver disease requiring antiviral therapy in 4 of 66 cases (6%) during a follow-up of 24 months.

The definition of a so-called “HBV-inactive carrier” is based on repeated and stable ALT normality and HBV-DNA <2000 IU/ml associated with normal findings at liver ultrasound (US). LB is an invasive test not recommended in these patients. TE is a noninvasive tool, easy, feasible, and reproducible, to investigate and follow up these patients [6, 7].

The use of combined scores of fibrosis in patients with HBV infection is controversial: for some authors, these tests could be helpful in the screening of patients with HBV infection [23, 24], but no studies have investigated their effectiveness in a cohort of putative inactive HBV carriers. Instead, our data suggested that noninvasive scores of fibrosis used (APRI test, Forns Index, and FIB4) do not allow to identify subjects with significant fibrosis (LS ≥7.5 kPa) nor individuals with high viral load (HBV-DNA ≥2000 IU/ml) in this setting. Therefore, these tests are not useful in the initial assessment of the putative inactive HBV carrier and in the following controls.

In the initial evaluation of the inactive HBV carrier, FibroScan® is mandatory, coupled with quantitative HBsAg determination, when available. Patients with HBV-DNA levels between 2000 and 20000 IU/ml, previously called “grey zone,” cannot be considered “tout court” inactive carriers, but for patients with active viral replication and thus with potentially progressive liver disease [7, 25], a more strict biochemical follow-up (every 3–6 months) and a TE every 12 months is recommended [7]. In fact, it could allow us to identify a “HBV false inactive carrier,” with an active liver disease as confirmed by a subsequent liver biopsy (Figure 1).

Another critical point of this study is the identification of other concomitant causes of liver disease in so-called inactive carriers. Already, Oliveri et al. in their study [20] identified among 188 patients a group of 17 (9%) as “inactive carriers with liver disease” having a significant liver stiffness. In these patients, causes other than viruses (metabolic, toxic, or unidentified) may produce liver disease or potentially contribute to its progression. In our follow-up group, these patients were about 5/51 (9.8%) (Table 5), in accordance with the data of the other authors. Therefore, false inactive carriers of the virus with cofactors of the disease should undergo, in addition to the initial screening for other causes of chronic liver disease (i.e., autoimmune, genetic, and toxic), a quantitative evaluation of steatosis either by ultrasound score of Hamaguchi [16] or controlled attenuation parameter (CAP) measurement if available [15], and the calculation of Fatty Liver Index [14] and the most used score of fibrosis (NAFLD Fibrosis Score, BARD) [17, 18]. It is in fact known that the cutoffs of FibroScan® vary according to the etiology of liver disease [11, 12], and therefore, it would be useful to propose studies evaluating patients with double etiology (viral and NAFLD) in order to find an algorithm applicable to FibroScan® for correction of the final result in such patients. The finding in our study population of some individuals with LS ≥7.5 could be due to fibrosis caused by previous hepatitis flares and to the presence of multifactorial liver disease. The availability of quantitative determination of HBsAg test as applicable to all HBsAg positive cases allows, hypothetically, to identify more accurately the patients with transcriptionally active cccDNA and thus able to develop progressive disease [25]. Although this information cannot be deduced from our observational study, the test was applied only in a subgroup of patients with higher probability of falsely inactive infection (HBV-DNA ≥2000 and ≤20000 IU/ml).

## 6. Conclusions

In conclusion, in our study, we confirmed that FibroScan® is a simple and reproducible method for noninvasive assessment of liver fibrosis in the so-called inactive carrier of HBV and is potentially very useful in clinical practice for the staging of these patients [7]. An optimal timing for the execution of FibroScan® in putative inactive HBsAg carriers is not yet determined, but probably on the basis of our data, testing repetition every 6–36 months (according to our flow chart) should rule out false inactive carriers with a potentially progressive liver disease.

## Figures and Tables

**Table 1:** Baseline characteristics of the included 125 patients.

Characteristics	Results
Patients, *n*	125
Age (years)	46.5 ± 13.8
Gender	
Male, *n* (%)	77 (61.6%)
Female, *n* (%)	48 (38.4%)
Citizenship	
Italians, *n* (%)	75 (60.0%)
Foreigners, *n* (%)	50 (40.0%)
ALT	0.59 ± 0.26
AST	0.64 ± 0.19
HBV-DNA	3747.46 ± 13883.25
HBV-DNA <2000 IU/ml, *n* (%)	91 (72.8%)
HBV-DNA ≥2000 IU/ml, *n* (%)	34 (27.2%)
HBV-DNA >20000 IU/ml, *n* (%)	4 (3.2%)
Liver stiffness (kPa)	5.55 ± 1.92
LS <7.5 kPa, *n* (%)	107 (85.6%)
LS ≥7.5 kPa, *n* (%)	18 (14.4%)
APRI	0.29 ± 0.11
Forns Index	4.15 ± 1.63
FIB4	1.16 ± 059

Continuous values are expressed as mean ± standard deviation (SD). ALT and AST are expressed as multiples of upper limit of normal.

**Table 2:** Characteristics of Italians versus foreigners among 125 patients.

Characteristics	Italians (*n* = 75)	Foreigners (*n* = 50)	*p*
Age (years)	53.1 ± 11.9	36.5 ± 10.2	<0.0001
ALT	0.53 ± 0.18	0.67 ± 0.33	<0.05
AST	0.59 ± 0.16	0.70 ± 0.21	<0.01
Liver stiffness (kPa)	5.59 ± 1.92	5.50 ± 1.94	Ns
LS < 7.5 kPa, *n* (%)	64 (85.3%)	43 (86.0%)	Ns
LS ≥ 7.5 kPa, *n* (%)	11 (14.7%)	7 (14.0%)	
HBV-DNA	3107.29 ± 8975.52	4707.72 ± 19092.72	Ns
HBV-DNA <2000 IU/ml, *n* (%)	58 (77.3%)	33 (66.0%)	Ns
HBV-DNA ≥2000 IU/ml, *n* (%)	17 (22.7%)	17 (34.0%)	

Continuous variables are expressed as mean ± standard deviation (SD). A *p* value <0.05 is considered significant (Wilcoxon test). ALT and AST are expressed as multiples of upper limit of normal. For categorical variables, a *p* value <0.05 is considered significant (Chi-squared test).

**Table 3:** Fibrosis score results according to LS among 125 patients.

Characteristics	LS <7.5 kPa	LS ≥7.5 kPa	*p*
	(*n* = 107)	(*n* = 18)	
APRI	0.28 ± 0.09	0.35 ± 0.18	Ns
Forns Index	4.14 ± 1.55	4.17 ± 2.06	Ns
FIB4	1.15 ± 0.60	1.21 ± 0.59	Ns

All values are expressed as mean ± standard deviation (SD). A *p* value <0.05 is considered significant (Wilcoxon test).

**Table 4:** HBV-DNA and liver stiffness in the subgroup of 66 patients during the follow-up.

Characteristics	Basal time	12 months	24 months	Change (24 months, basal time)	
				% change	*p* value
HBV-DNA	561.80 ± 620.01	5200.27 ± 18666.56	5202.76 ± 19597.25	826.1	<0.001
HBV-DNA <2000 UI/ml, *n* (%)	66 (100%)	51 (77.3%)	56 (84.8%)	−15.2	—
HBV-DNA ≥2000 UI/ml, *n* (%)	0 (0%)	15 (22.7%)	10 (15.2%)	100.0	
Liver stiffness (kPa)	5.80 ± 2.29	5.66 ± 1.43	5.67 ± 1.62	−2.2	Ns
LS < 7.5 kPa, *n* (%)	50 (75.8%)	54 (81.8%)	54 (81.8%)	8.0	Ns
LS ≥ 7.5 kPa, *n* (%)	16 (24.2%)	12 (18.2%)	12 (18.2%)	−25.0	

Continuous variables are expressed as mean ± standard deviation (SD). A *p* value <0.05 is considered significant (Wilcoxon paired test). For categorical variables, a *p* value <0.05 is considered significant (McNemer's test).

**Table 5:** Relation between HBV-DNA and liver stiffness in the subgroup of 66 patients during the follow-up.

Characteristics	HBV-DNA <2000 UI/ml	HBV-DNA ≥2000 UI/ml	*p* value
Basal time			
Liver stiffness (kPa)	5.80 ± 2.29	—	—
LS < 7.5 kPa, *n* (%)	50 (100%)	0 (0%)	—
LS ≥ 7.5 kPa, *n* (%)	16 (100%)	0 (0%)	

12 months			
Liver stiffness (kPa)	5.33 ± 1.22	6.79 ± 1.55	<0.001
LS < 7.5 kPa *n* (%)	47 (87.0%)	7 (13.0%)	<0.001
LS ≥ 7.5 kPa *n* (%)	4 (33.3%)	8 (66.7%)	

24 months			
Liver stiffness (kPa)	5.30 ± 1.43	7.69 ± 1.07	<0.0001
LS < 7.5 kPa, *n* (%)	51 (94.4%)	3 (5.6%)	<0.0001
LS ≥ 7.5 kPa, *n* (%)	5 (41.7%)	7 (58.3%)	

Continuous variable are expressed as mean ± standard deviation (SD). A *p* value <0.05 is considered significant (Wilcoxon test). For categorical variables, a *p* value <0.05 is considered significant (Fisher's test).

## Data Availability

All data generated or analyzed during this study are included within this article.
